# Chronic upper airway obstruction induces abnormal sleep/wake dynamics in juvenile rats

**DOI:** 10.1186/1471-2202-15-S1-P210

**Published:** 2014-07-21

**Authors:** Gideon Gradwohl, Nilly Berdugo-Boura, Yael Segev, Ariel Tarasiuk

**Affiliations:** 1 Sleep-Wake Disorders Unit, Soroka University Medical Center and Department of Physiology, Faculty of Health Sciences, Ben-Gurion University of the Negev, 84110, Israel; 2 Shraga Segal Department of Microbiology and Immunology, Faculty of Health Sciences, Ben-Gurion University of the Negev, 84110, Israel; 3 Unit of Biomedical Engineering, Department of Physics, Lev Academic Center, Jerusalem, 9116001, Israel

## 

Traditional scoring of sleep provides little information about the process of transitioning between vigilance-states.

We hypothesized that chronic upper airway obstruction (UAO) will lead to sleep instability in the absence of frank obstructed apneas or hypopneas.

Using an approach similar to previous reports [1,2] defined a 2-dimensional (2D) state space technique (SST) using 2 spectral amplitude ratios calculated by dividing integrated spectral amplitudes at selected frequency bands. We calculated two spectral amplitude ratios by integrating the spectral amplitude over specific frequencies: 0.5-20/0.5-40 Hz for ratio 1 and 0.5-4/0.5-9 Hz for ratio 2. Each second of this ratio was mapped into the 2D state space, ratio 2 vs. ratio 1 (Figure 1). State space analysis yields wake at low ratio 1 values and SWS at high ratio 1 values. We used the SST to explore whether the abnormal sleep in UAO reflects sleep instability, faster movements, and abnormal transitions between vigilance-states.

**Figure 1:**
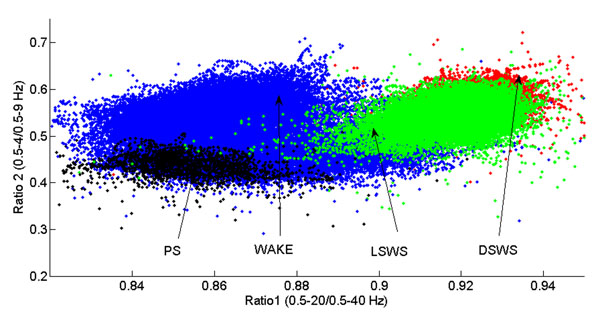
Spontaneous sleep in rats. Values of wake (W), light slow wave sleep (LSWS), deep slow wave sleep (DSWS) and paradoxical sleep (PS) are shown.

We analyzed whole day electroencephalography sleep/wake recordings of UAO and sham control juvenile Sprague-Dawley rats using SST. SST is a non-categorical approach allowing quantitative and unbiased examination of sleep/wake states and state transitions. Measurements of sleep/wake recordings were performed at baseline and following administration of ritanserin (5-HT2 receptor antagonist) the next day to stimulate sleep.

UAO rats spent less time in deep (delta-rich) slow wave sleep (SWS) and near transition zones between states. Sleep state transitions were more frequent and rapid in UAO rats, indicating that obstructed animals have more regions where vigilance-states are unstable. The number of microarousals and trajectories from light SWS to wake and vice versa increased in the UAO group. Ritanserin consolidated sleep in both groups by decreasing the number of microarousals and trajectories between wake to light SWS, and increasing deep SWS in UAO.

## Conclusions

Traditional sleep scoring in 10-second epochs reveals little information about the process of transitioning between states, as cortical activity and behavior can change quite rapidly in rodents; the SST analysis provides second-by-second resolutions. Also, conventional scoring simply identifies discrete states, so it can overlook important variations within states, such as the distinctions between light and deep SWS or between sleep and wake states. We present evidence that SST enables visualization of vigilance-state transitions and velocities that were not evident by traditional scoring methods. These observations provide new insight on abnormal sleep and highlight the usefulness of SST in understanding the role of UAO on sleep dynamics.
